# Altered Faecal Microbiota Composition and Structure of Ghanaian Children with Acute Gastroenteritis

**DOI:** 10.3390/ijms24043607

**Published:** 2023-02-10

**Authors:** Emmanuel Kofi Quaye, Raymond Lovelace Adjei, Abiola Isawumi, David J. Allen, J. Gregory Caporaso, Osbourne Quaye

**Affiliations:** 1 West African Centre for Cell Biology of Infectious Pathogens (WACCBIP), Department of Biochemistry, Cell and Molecular Biology, University of Ghana, Accra P.O. Box LG 54, Ghana; 2 Council for Scientific and Industrial Research (CSIR)-Animal Research Institute, Accra P.O. Box AH 20, Ghana; 3 Department of Infection Biology, London School of Hygiene and Tropical Medicine, London WC1E 7HT, UK; 4 Vaccine Centre, London School of Hygiene and Tropical Medicine, London WC1E 7HT, UK; 5 Centre for Applied Microbiome Science, Pathogen and Microbiome Institute, Northern Arizona University, Flagstaff, AZ 86011, USA; 6 Department of Biological Sciences, Northern Arizona University, Flagstaff, AZ 86011, USA

**Keywords:** acute gastroenteritis, children, faecal microbiota, bacteria, disease, pathogen, correlation network

## Abstract

Acute gastroenteritis (AGE) is a disease of global public health importance. Recent studies show that children with AGE have an altered gut microbiota relative to non-AGE controls. Yet, how the gut microbiota differs in Ghanaian children with and without AGE remains unclear. Here, we explore the 16S rRNA gene-based faecal microbiota profiles of Ghanaian children five years of age and younger, comprising 57 AGE cases and 50 healthy controls. We found that AGE cases were associated with lower microbial diversity and altered microbial sequence profiles relative to the controls. The faecal microbiota of AGE cases was enriched for disease-associated bacterial genera, including *Enterococcus*, *Streptococcus,* and *Staphylococcus*. In contrast, the faecal microbiota of controls was enriched for potentially beneficial genera, including *Faecalibacterium*, *Prevotella*, *Ruminococcus*, and *Bacteroides*. Lastly, distinct microbial correlation network characteristics were observed between AGE cases and controls, thereby supporting broad differences in faecal microbiota structure. Altogether, we show that the faecal microbiota of Ghanaian children with AGE differ from controls and are enriched for bacterial genera increasingly associated with diseases.

## 1. Introduction

Despite significant reductions in childhood morbidity and mortality over the past decades, acute gastroenteritis (AGE) remains a major burden [1]. Almost 525,000 children die annually from over 1.7 billion cases of AGE [2]. The incidence, morbidity, and mortality associated with AGE are highest in developing countries [3]. AGE is characterised by the passage of at least three loose/watery stools per day, with abdominal pain, fever, and vomiting [2]. Severe dehydration used to be the main cause of AGE-associated deaths globally [4]. However, deaths are increasingly linked with infectious microbial agents, including viruses (rotavirus, norovirus, sapovirus, enteric adenovirus) [5], bacteria (*Campylobacter*, *Escherichia*, *Shigella*, *Vibrio cholerae*) [5], and eukaryotic parasites (*Cryptosporidium*, *Giardia*, *Entamoeba*) [5]. These pathogens are transmitted through the faecal–oral route, where they colonise and cause AGE. Infection occurs through the consumption of contaminated food and water and sometimes contact with an infected individual [2].

Lining the human gut are trillions of microbes, including bacteria, fungi, archaea, viruses, and eukaryotes, collectively known as the gut microbiota [6]. The gut microbiota is present at birth and increases in number and community complexity over the first few years of life [7,8]. Members of the gut microbiota interact at the physical and chemical levels in defence of their niche and compete for nutrient supply [9,10]. The benefits derived from gut microbiota include food digestion; protection against pathogen adhesion and colonisation; and contribution to immune, metabolic, and neurobiological development [6,11,12]. Consequently, alteration of the homeostatic balance of the gut microbiota in childhood increases the risk of developing diseases, including allergies, autoimmunity, inflammatory bowel diseases, diabetes, and obesity [6,13].

AGE is associated with gut microbiota alterations, irrespective of aetiological agents, albeit to different extents, with variable microbial features identified [14,15,16,17,18]. Faecal samples from AGE cases are associated with the presence of pathogens and/or microbes with increased pathogenic potential in higher abundance [16]. The incidence of AGE in children and its association with gut microbiota alterations result in gut barrier damage and reduced nutrient absorptive capacity [19]. This highlights the importance of studying gut microbiota profiles in AGE.

A recent study showed differences in the faecal microbiota profiles of Ghanaian patients (children, adolescents, and adults) with viral gastroenteritis relative to healthy adult controls [20]. While the findings of this study support the association of AGE cases with microbiota alterations, how these observations apply specifically in children, who suffer the highest burden of AGE, remains unclear. This study explores the faecal microbiota of Ghanaian children ≤ 5 years with and without AGE.

## 2. Results

### 2.1. Demographic and Clinical Characteristics of Study Participants

A total of 107 faecal samples from 57 AGE cases and 50 non-AGE “healthy controls” were used for this study (Table 1). The mean age of AGE cases was lower than healthy controls. No differences were found in the proportions of sex and rotavirus vaccination status between AGE cases and healthy controls. A higher proportion of AGE cases reported vomiting and fever as symptoms. Whereas a higher proportion of AGE cases were fed artificial milk, a higher proportion of healthy controls were fed family meals (meals prepared and shared by the whole family). A comparable proportion of AGE cases and healthy controls were breastfed or formula-fed.

### 2.2. AGE Cases Have Lower Alpha Diversity and Distinct Beta Diversity Profiles

**Figure 1:**
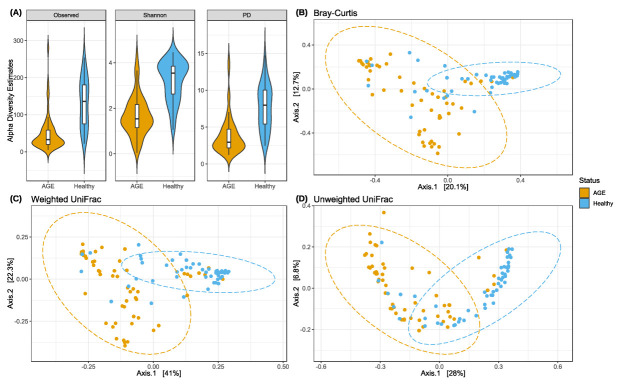
AGE cases are associated with lower alpha diversity and distinct beta diversity relative to healthy controls. (**A**) Violin plot of alpha diversity metrics. Left to right: Observed ASVs (*p* < 0.0001, Wilcoxon rank sum test), Shannon (*p* < 0.0001, Wilcoxon rank sum test), and phylogenetic diversity (PD) (*p* < 0.0001, Wilcoxon rank sum test). Violin plots show the kernel probability density plots of individual alpha diversity estimates. Boxplots show the median (middle line: 50th percentile), first (bottom: 25th percentile), and third quartiles (top: 75th percentile), and whiskers as 1.5 times the interquartile range. Principal coordinates analysis (PCoA) plots of (**B**) Bray–Curtis dissimilarity (*p* < 0.001, PERMANOVA R^2^ = 0.09) and (**C**) Weighted (*p* < 0.001, PERMANOVA R^2^ = 0.19) and (**D**) Unweighted UniFrac metrics (*p* < 0.001, PERMANOVA R^2^ = 0.12). Percentage variation explained on axes 1 and 2 are shown. Ellipses show the 95% confidence interval for the variation within each group. Each dot represents a faecal microbiota sample from a different individual.

Quality filtering and removal of suspected contaminants and uncharacterised taxa resulted in 2897 unique amplicon sequence variants (ASVs), covering 18 phyla, 37 classes, 65 orders, 107 families, and 216 genera. Three metrics were used to estimate the within-sample (alpha) diversity after rarefying to an ASV sequence count of 10,000 (Figure 1A). AGE cases had significantly lower faecal microbiota richness (observed ASVs), Shannon diversity, and phylogenetic diversity estimates compared with healthy controls. Significant differences in alpha diversity estimates persisted with age, breastfeeding, family meal, formula, and artificial milk intake, as covariates in simple linear models (Supplementary Table S1). High dimensional faecal microbial 16S rRNA gene sequence profiles were consistent with previous findings, with observed significant differences based on Bray–Curtis dissimilarity (Figure 1B) and weighted (Figure 1C) and unweighted UniFrac (Figure 1D) metrics. Participant age and breastfeeding status were small but significant explanatory covariates, whereas family meal, formula, and artificial milk intake were nonsignificant (Supplementary Table S2). These suggest that the faecal microbiota structure of AGE cases was distinct from healthy controls.

### 2.3. Taxonomic Profiles Differ between AGE Cases and Healthy Controls

**Figure 2:**
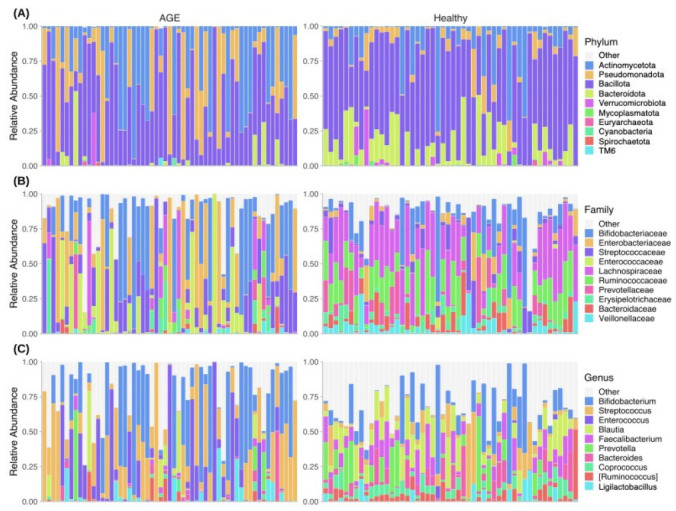
Faecal microbial sequence profiles of AGE cases and healthy controls. Relative abundance of (**A**) Phylum-, (**B**) Family-, and (**C**) Genus-level features. Samples are grouped according to status (AGE cases or healthy controls). Each bar represents an individual faecal microbial sequence profile. Top 10 most dominant taxa are shown, with the remaining collapsed under “Other”.

The dominant phyla present in the faecal microbial sequence profiles were *Bacillota*, *Actinomycetota*, *Pseudomonadota*, *Bacteroidota*, and *Verrucomicrobiota* (Figure 2A). AGE cases had lower relative abundances of *Bacteroidota*, *Bacillota*, *Mycoplasmatota*, and *Verrucomicrobiota* (Figure 2A). The dominant family included *Bifidobacteriaceae*, *Enterobacteriaceae*, *Lachnospiraceae*, *Streptococcaceae*, and *Enterococcaceae* (Figure 2B). Family features with high relative abundance in AGE cases included *Corynebacteriaceae*, *Enterococcaceae*, *Micrococcaceae*, and *Streptococcaceae*. Further, there were differences in the relative abundance of genera between AGE cases and healthy controls. *Atopobium*, *Enterococcus*, *Rothia*, and *Streptococcus* genera were higher in AGE cases, while *Akkermansia*, *Bacteroides*, *Dialister*, *Faecalibacterium*, and *Prevotella* were higher in healthy controls (Figure 2C). These observations suggest there are differences between the taxonomic composition of AGE cases and healthy controls.

### 2.4. Shared and Unique Core Genera between AGE Cases and Healthy Controls

**Figure 3:**
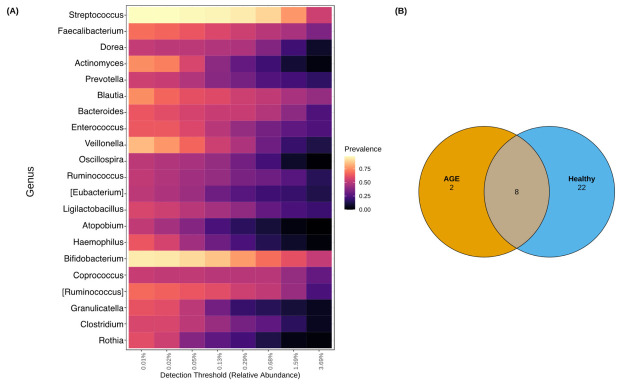
Core genera of AGE cases and healthy controls. (**A**) Heatmap of core genera across samples of the two groups. Genus names are shown. (**B**) Venn diagram of the number of core genera identified within AGE cases and healthy controls. Core genera were identified using a 50% prevalence cut-off and an abundance cut-off of 0.01% (0.0001).

Twenty-one core genera were identified (at a minimum detection threshold of 0.01% and 50% prevalence) (Figure 3A). These included *Streptococcus*, *Faecalibacterium*, *Bacteroides*, *Bifidobacterium*, *Blautia, Enterococcus*, and *Veillonella*. *Streptococcus*, *Bifidobacterium*, *Veillonella*, *Blautia*, and *Actinomyces* had the highest overall prevalence. Group-specific core genera revealed 2 unique genera to AGE cases, 22 to healthy controls, and 8 overlapped between both groups (Figure 3B; Supplementary Table S3). *Atopobium* and *Rothia* were unique to AGE cases, and *Faecalibacterium*, *Bacteroides*, *Prevotella*, *Coprococcus*, *Clostridium*, and *Dialister* were part of the 22 genera unique to healthy controls. The overlapping genera were *Bifidobacterium*, *Blautia*, *Enterococcus*, *Veillonella*, *Granulicatella*, *Actinomyces*, *Streptococcus*, and *Ligilactobacillus*.

### 2.5. Faecal Microbial Sequence Profiles Based on Differential Abundance Testing

**Figure 4:**
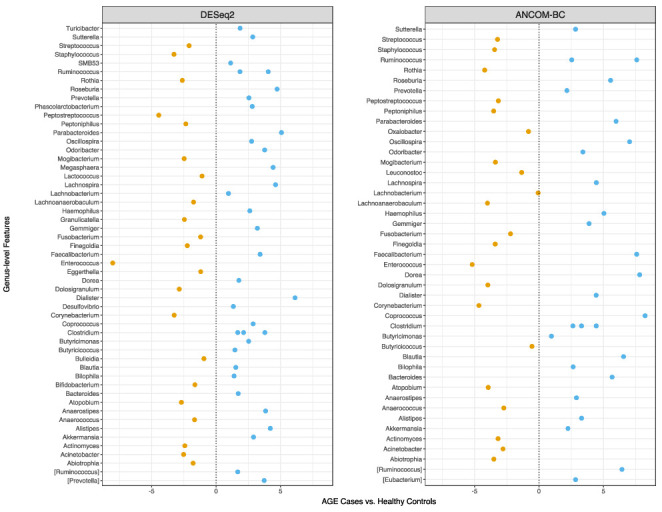
Differential abundance testing identifies genera with high abundance between AGE cases and healthy controls. DESeq2 (**left**) and ANCOM-BC (**right**) were used for differential abundance testing. Genera that passed multiple test correction (Benjamini and Hochberg’s FDR < 0.05) are shown. Dots represent the estimated effect size distribution as log2 (fold-change) and *W* for DESeq2 and ANCOM-BC, respectively. Negative values represent genera enriched in AGE cases and positive values represent genera enriched in healthy controls. Differential abundance testing identifies genera with high abundance between AGE cases and healthy controls. DESeq2 (**left**) and ANCOM-BC (**right**) were used for differential abundance testing. Genera that passed multiple test correction (Benjamini and Hochberg’s FDR < 0.05) are shown. Dots represent the estimated effect size distribution as log2 (fold-change) and *W* for DESeq2 and ANCOM-BC, respectively. Negative values represent genera enriched in AGE cases and positive values represent genera enriched in healthy controls.

Overabundant genera between AGE cases and healthy controls were identified after prefiltering to include only features observed in at least 10% of samples. By using DESeq2, 56 differentially abundant bacteria were identified (Figure 4), and these were annotated to 53 different genera. Of these, ~42% (22/53) of the genera were enriched in AGE cases, and these included *Enterococcus*, *Peptostreptococcus*, *Staphylococcus*, *Corynebacterium*, *Dolosigranulum*, *Atopobium*, *Granulicatella, Rothia, Mogibacterium*, and *Streptococcus*. In contrast, the genera enriched in healthy controls included *Dialister*, *Roseburia*, *Ruminococcus*, *Clostridium*, *Faecalibacterium*, *Akkermansia*, and *Prevotella*.

Additionally, we confirmed the output of DESeq2 with ANCOM-BC, a tool that is robust to compositionality. ANCOM-BC identified 45 genera as differentially abundant between the two groups (Figure 4). Notably, the enrichment of *Enterococcus*, *Staphylococcus*, *Corynebacterium*, *Mogibacterium*, *Rothia*, *Dolosigranulum*, and *Peptostreptococcus*, in AGE cases, corresponded with the output from DESeq2, albeit with different effect size estimates. Similar observations were made for genera enriched in healthy controls, such as *Prevotella*, *Akkermansia*, *Faecalibacterium*, *Roseburia*, and *Ruminococcus*. Thus, the faecal microbial sequence profiles of AGE cases and controls were enriched for different bacteria based on differential abundance testing.

### 2.6. Selbal Identifies Balances Associated with AGE Cases and Healthy Controls

**Figure 5:**
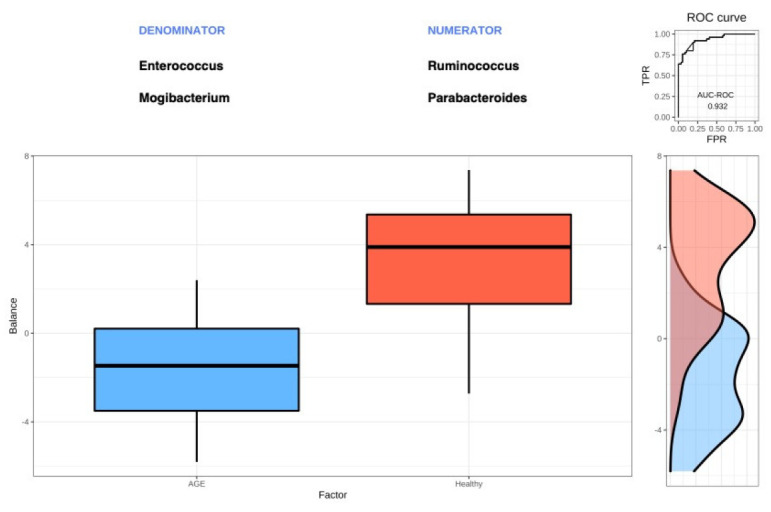
Global balance for AGE cases and healthy controls. The two groups of genera that form the global balance are shown at the top of the boxplot, which shows the distribution of balance scores. Boxplots show the median (middle line: 50th percentile), first (bottom: 25th percentile), and third quartiles (top: 75th percentile), and whiskers as 1.5 times the interquartile range. The right section of the figure shows the AUC-ROC curve value of 0.932 and the data density plot for each group.

Two groups of balances (as numerator and denominator) discriminating between AGE cases and healthy controls were determined using Selbal [21], a forward-selection method. Four genera were identified as optimal after cross-validation, with a mean accuracy (area under the curve [AUC]) of 0.848 (Figure 5; Supplementary Figure S1). The accuracy value (AUC) of 0.848 provided from cross-validation was lower than the AUC-ROC (receiver-operator characteristic) curve of 0.932 (top right of Figure 5), as the latter is an overestimation measured on the same data for model building. The four genera were *Ruminococcus* and *Parabacteroides* (numerator: most associated with healthy controls) and *Enterococcus* and *Mogibacterium* (denominator: most associated with AGE cases). These results further support the outcome of the differential abundance testing.

### 2.7. Network Analysis further Reveals Differences in Faecal Microbiota Structure

**Figure 6:**
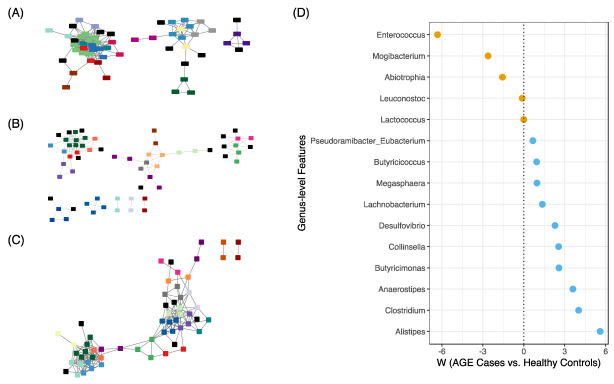
Correlation network analysis of faecal microbiota. Correlation networks for (**A**) All groups, (**B**) AGE cases, and (**C**) Healthy controls. Module memberships identified by SCNIC are shaded with colours other than black (for instance, shared membership under module-0 is shaded light green on panel **A**). Nodes (with rectangular/square shapes) represent genera, and edges represent correlations with an R-value greater than 0.35. (**D**) Differential abundance testing with ANCOM-BC. Genera that passed multiple test correction (Benjamini and Hochberg’s FDR < 0.05) are shown. Dots with negative *W* values represent genera enriched in AGE cases, while positive values represent genera enriched in healthy controls.

High-level insights into the faecal microbiota structure of AGE cases and healthy controls were determined by inferring a correlation network on genus-level features. The resulting network across all samples had 65 nodes and 213 edges and was summarised into 19 modules (module-0 to module-18), the most significant of which included nine genera (Figure 6A; Supplementary Table S4). We inferred the correlation network structure between AGE cases and healthy controls separately and identified a unique set of modules and memberships. AGE cases had a less tightly connected network, with 61 nodes, 122 edges, and 18 modules (Figure 6B; Supplementary Table S4), while controls had 59 nodes, 205 edges, and 19 modules (Figure 6C; Supplementary Table S4). By applying ANCOM-BC to the feature table produced from SCNIC, we identified 15 genera associated with AGE cases and healthy controls (Figure 6D), the majority of which were also individually significant without SCNIC (Figure 4). *Enterococcus* had the highest effect size and was, together with *Mogibacterium*, *Abiotrophia*, *Leuconostoc*, and *Lactococcus*, associated with AGE cases. Conversely, the 10 genera associated with healthy controls included *Alistipes*, *Clostridium*, *Anaerostipes*, and *Butyricimonas*. Thus, the faecal microbiota structure of AGE cases differed from healthy controls based on correlation network inference.

## 3. Discussion

The period of childhood from birth to 5 years is the most important for immune and metabolic imprinting by gut microbes and their functional products [22]. Children suffer several bouts of infections, partly due to an immature immune system or increased exposure to pathogens in the environment. Most of these pathogens are transmitted through the faecal–oral route, where they colonise and establish infections in the gastrointestinal tract (GIT). Infection in the GIT is akin to most diarrhoeal diseases and is associated with the passage of loose/watery stools and gut microbiota alterations. However, it remains unclear how the gut microbiota differs in Ghanaian children with and without AGE. We profiled the faecal microbiota of Ghanaian children with and without AGE using 16S rRNA gene sequencing.

Lower alpha diversity observed in AGE cases is characteristic of many GIT diseases, including AGE [16,23] and functional gastrointestinal disorders [24], which may result from diverse factors. These factors include the “gushing” reaction that extrudes microbes from the gut [23], aberrant inflammatory response against invading agent(s) [6,11], and transiently aerobic gut conditions, which kill obligate anaerobes and encourages the expansion of facultative anaerobes [17]. Moreover, the lower mean age of AGE cases than healthy controls contributed to the observed lower alpha diversity, as age and its associated changes in dietary choices (cessation of breastfeeding/introduction of solid meals) are known factors that drive gut microbiota maturation [13,25,26]. Importantly, the lower mean age for AGE cases compared to controls was driven by the relatively high disease burden of AGE in Ghanaian children younger than 24 months, increasing the likelihood of hospitalisation and recruitment into studies with hospital-based sampling design [27,28]. While lower alpha diversity is linked with AGE cases, the effect of medication(s) likely administered to treat AGE, which was not reported in this study, could further have reduced overall microbial diversity [29,30]. Loss of microbial diversity drove the observed differences in beta diversity estimates, suggesting that the representation (presence or absence), abundance of taxa (dominant and rare), and phylogenetic relationships were different [31,32].

The observed differences in microbial sequence profiles at the phylum, family, and genus levels suggest that AGE was associated with changes in taxonomic composition [16,33]. The composition of the taxonomic core was similar to previous findings in Ghanaian children [34,35]. This relates to microbes that may be inherited from maternal and environmental sources [7], those unaffected by differing environmental exposures, and those important for the host’s immune, nutritional, and metabolic development and function [36].

AGE cases were enriched for genera increasingly linked with infections of public health importance due to their intrinsic and acquired virulence, biofilm-forming, and antimicrobial resistance properties. These findings reflect the selective advantage available to these taxa and the increased chance for gut surface colonisation provided through reduced microbial diversity [37]. *Enterococcus* sp. was one notable genus with the highest effect size of enrichment. Enterococci are linked with bacterial infections in paediatric patients, immunocompromised individuals, and mouse models [38,39,40]. Enterococci have previously been observed to be enriched in the faecal microbiota of Vietnamese paediatric patients and Bangladeshis with infectious AGE [15,17]. In addition, several genera of the upper GIT dominated the enriched faecal microbiota fraction of AGE cases, as shown previously [16,17,41], thereby signifying an increased transmission of microbes from the mouth to the gut [42]. Some examples of these include *Rothia*, *Actinomyces*, *Atopobium*, *Mogibacterium*, *Peptostreptococcus*, and *Fusobacterium*. Transmission of these bacteria could possibly go beyond passive to active acclimatisation to the gut environment, as planktonic forms or biofilms [42]. We posit that one reason driving the enrichment of oral taxa in AGE cases, aside from the low microbial diversity, could be due to their adaptation to, and use of, elevated nitrogen and oxygen species (e.g., nitric oxide) [43,44,45]. These, coupled with a loss of intestinal barrier integrity, could increase the risk of bacterial translocation and infection [46].

As expected, the faecal microbiota of the controls was enriched for potentially beneficial bacteria linked with good health. These included *Faecalibacterium*, *Anaerostipes*, *Dialister*, *Ruminococcus*, *Bacteroides*, *Akkermansia*, *Coprococcus*, and *Prevotella*. As well as producing beneficial metabolites, these bacteria metabolise complex plant polysaccharides to produce short-chain fatty acids (SCFAs), which have immunoregulatory and physiological functions [47,48,49]. For instance, *Faecalibacterium* exerts its anti-inflammatory activities by stimulating the production of interleukin 10 (IL-10) and limiting the production of tumour necrosis factor (TNF) [12]. Further, the depletion of SCFA-producing bacteria may explain the loss of water, electrolyte absorption, and reduced metabolism by enterocytes, further increasing the severity of AGE [17,50].

Microbial interaction mediates communication and allows for a coordinated response to environmental cues. A sparsely connected network in AGE cases, compared with healthy controls, is consistent with previous findings [16] and is intricately linked with lower microbial diversity, supporting theories linking ecosystem diversity to community balance and stability [16,51,52]. Module memberships between the two groups were unique from those identified previously [16] and were made up of both potentially beneficial and disease-associated genera. This could point to potential roles to restore or maintain ecosystem stability. The dominance of oral taxa in modules suggests that they share the same niche and may form polymicrobial biofilms that allow them to survive the harsh gut environment and are likely to interact at the molecular level [17,53]. Network-based differential abundance tests further confirmed the increased abundance of disease-associated genera in AGE cases and the depletion of potentially beneficial bacteria in AGE cases.

The genera identified in this study are suggestive but not necessarily pathogens, as we did not present strain-level features or prove a causal role for these in AGE. The findings in this study are limited by the lack of longitudinal samples, which makes it impossible to determine temporal faecal microbiota dynamics before, during, and after AGE. Faecal samples were used as a proxy for gut samples because they are the most commonly used non-invasive means of sampling and may not fully represent the gut microbiota profile. New AGE cases could not be assessed and recruited; therefore, archived samples were used in this study. Furthermore, details on the screening of faecal samples for enteropathogens were not reported. The study lacked extensive information on potential confounders, such as antibiotic use, underlying medical conditions, ethnicity, birth mode, and nutrition-specific questionnaire data to capture diet components and their estimated quantities.

In conclusion, the study showed differences in faecal microbiota profiles between Ghanaian children with and without AGE. The faecal microbiota of AGE cases was dominated by disease-associated bacterial genera, most of which were notable members of the upper GIT and was depleted in beneficial bacteria linked with good health. Finally, whole microbial community network characteristics differed between AGE cases and controls. The findings could have implications for the outcome of AGE.

## 4. Materials and Methods

### 4.1. Study Design and Participant Recruitment Criteria

The study was a cross-sectional case–control study in Greater Accra Region, Ghana. A case was defined as a child who presented with AGE. Archival faecal samples from children admitted or presented to the hospital with AGE (three or more loose or watery stools per day) were included in the study as AGE cases. Intake of antibiotics or other medication was not reported for AGE cases. Children who were otherwise healthy at the time of sample collection, without AGE (diarrhoea), and had not taken antibiotics at least 30 days before sampling, based on the parental description of child health and clinical history, were sampled as healthy controls. All children were aged 5 years and below, with the minimum age considered at 1 month. Children whose parents/guardians did not consent to the study, were over 5 years old (>60 months), had been on antibiotics for less than 30 days before sampling (healthy controls only), were sick or with a recent case of diarrhoea (less than 30 days before sampling), and those who had been on probiotic supplements were excluded.

### 4.2. Sample Size, Sample Collection, and Processing

A power calculation to estimate the expected effect size was not predetermined. Nonetheless, we considered at least 40 faecal samples for each study group to be sufficient, based on previously published studies [54,55,56]. Faecal samples were collected into sterile transparent containers fitted with a spatula, stored temporarily in a refrigerator/freezer on-site, and transported on ice to the laboratory for storage (−80 °C). Five faecal samples were transported to the laboratory under ambient temperature within 30 min of collection. Archival AGE faecal samples were retrieved from storage (−20 °C or −80 °C).

### 4.3. DNA Extraction and 16S rRNA Gene Amplicon Sequencing

Genomic DNA was extracted from 107 faecal samples (approximately 0.19 ± 0.08 g), 6 extraction blanks as negative controls, and 2 mock communities of microbes (ZymoBIOMICS™ Microbial Community Standard and ZymoBIOMICS™ Microbial Community Standard II (Log Distribution), freely provided by Zymo Research Corporation, California, USA) using the DNeasy Powerlyzer PowerSoil kit (Qiagen, Hilden, Germany). Except for preheat treatment at 65 °C for 10 min and at 95 °C for 5 min, as well as mechanical disruption for 5 min, all extraction steps followed the manufacturer’s protocol. Genomic DNA purity and yield were quantified with the NanoDrop Lite spectrophotometer (Thermo Fisher Scientific, Waltham, Massachusetts, USA). Genomic DNA samples were shipped on dry ice for sequencing by the Environmental Sample Preparation and Sequencing Facility (ESPSF) at the Argonne National Laboratory, USA. Genomic DNA samples were subjected to high-throughput sequencing of the 16S rRNA gene [57]. Briefly, the V4 region was PCR amplified using the 515F/806R primer pair [58,59], and pooled amplicons were sequenced on the Illumina MiSeq (Illumina, Inc., San Diego, CA, USA), using 251 bp × 12 bp × 251 bp customised sequencing primers and procedures.

### 4.4. 16S rRNA Gene Sequence Processing

Paired-end FASTQ sequence files [60] were imported into QIIME 2 (version 2021.4) [61] and demultiplexed using the q2-demux plugin. Reads were quality-filtered, trimmed (forward reads at 240, reverse reads at 200), merged, and denoised to amplicon sequence variants (ASVs) using DADA2 through the q2-dada2 plugin [62]. The q2-fragment-insertion plugin [63] was used to construct a reference-based phylogenetic tree of ASVs using the Greengenes 13_8 (99%) database [64]. Fragments outside the insertion tree were filtered out because they either were erroneous or too distantly related to sequences in the reference tree [65]. Taxonomy was assigned using the q2-feature-classifier plugin’s scikit-learn naïve Bayes classifier [66] trained against the Greengenes database trimmed to include only the 515F/806R V4 region [67]. New names for the rank of bacterial phylum and the genus *Lactobacillus* were included [68]. ASVs misclassified as *Alloiococcus* instead of *Dolosigranulum* by Greengenes, as shown previously [69,70], were manually corrected.

QIIME 2 files were imported into R as a *phyloseq* object [71] using *qiime2R* (https://github.com/jbisanz/qiime2R; accessed on 25 July 2021). Additionally, *decontam* [72] was used to remove contaminants using the prevalence of ASVs identified in sequenced negative controls at a stringent classification threshold of 0.5. Uncharacterised and unassigned phyla were removed. Reads were rarefied to 10,000 to account for varying sequence counts prior to ecological diversity estimation (one AGE case sample was eliminated because of low read count) [73]. Alpha diversity was estimated on ASV data using the richness (Observed ASVs), Shannon, and Faith’s Phylogenetic Diversity (PD) metrics. Differences in alpha diversity measures based on categorical variables were tested using the Wilcoxon rank sum test. Beta diversity was estimated on ASV data using the Bray–Curtis dissimilarity index [74] and weighted and unweighted UniFrac distances [75,76]. Beta diversity was visualised using principal coordinates analysis (PCoA) plots. Significant differences in faecal microbiota structure between AGE cases and healthy controls were tested using the permutational multivariate analysis of variance (PERMANOVA) test implemented in the adonis function of the *Vegan* R package [77] with 999 permutations [78].

The core microbiota (defined as genera with at least 0.01% relative abundance and 50% prevalence) were determined using the *microbiome* R package [79]. Core microbiota in AGE cases only and healthy controls only were determined, as previously stated. DESeq2 [80] and ANCOM-BC [81] R packages were used for differential abundance testing, based on recommendations from [82], after filtering out genus-level features present in less than 10% of samples. The Selbal R package [21] was used to identify the two groups of taxa that significantly discriminate between AGE cases and controls, with five-fold cross-validation and 20 iterations. Selbal is different from the two differential abundance testing methods previously indicated, as it does not rely on FDR and power; however, it determines the best and most highly associated sparse model between AGE cases and healthy controls. We built a correlation network and detected and summarised modules from genus-level features using the Sparse Cooccurrence Network Investigation of Compositional data (SCNIC) through the q2-SCNIC plugin [83]. Prefiltering and inference of correlations were completed with the Sparse Correlations for Compositional data (SparCC) correlation metric [84] at a minimum R-value threshold of 0.35. ANCOM-BC was applied to the feature table generated by SCNIC across all samples. Network files were exported and visualised with Cytoscape [85], and Inkscape 1.1 (https://inkscape.org; accessed on 15 September 2021) was used to format correlation network figures.

### 4.5. Statistical Analysis

Demographic and clinical data were processed in R statistical software [86] through RStudio (1.4.1717). Categorical data were analysed by Pearson’s chi-squared (χ2) test. The age of participants was tested using the Wilcoxon rank sum test after the Shapiro–Wilk normality test for non-normal distribution was performed. Statistical significance was considered at *p* < 0.05. Where appropriate, *p*-values were adjusted for multiple comparisons using Benjamini and Hochberg’s FDR correction [87] unless otherwise stated. The R packages used for downstream data manipulation and visualisation included tidyverse suite [88], ggplot2 [89], viridis [90], RColorBrewer [91], Fantaxtic [92], microbiome [79], microbiomeutilities [93], eulerr [94], ggpubr [95], showtext [96], magrittr [97], broom [98], Hmisc [99], knitr [100], biomformat (https://github.com/joey711/biomformat), and scales [101].

## Figures and Tables

**Table 1:** Characteristics of study participants.

Number, *n*	AGE Cases	Healthy Controls	*p*-Value
	57	50	
Age (Months)			<0.0001
Mean ± SD	15.6 ± 14.2	29.6 ± 16.0	
(Range)	(1.5–60.0)	(5.0–59.0)	
Sex (%)			0.9884
Female	25 (43.9)	22 (44.0)	
Male	32 (56.1)	28 (56.0)	
Vomiting (%)			<0.0001
Yes	30 (52.6)	0 (0)	
No	23 (40.4)	50 (100)	
Missing *	4 (7.0)	-	
Fever (%)			<0.0001
Yes	44 (77.2)	0 (0)	
No	13 (22.8)	50 (100)	
Mode of Feeding, Yes (%)			
Breastfeeding	19 (33.3)	16 (32.0)	0.8834
Artificial Milk	12 (21.1)	2 (4.0)	0.0091
Formula	0 (0)	2 (4.0)	0.1274
Family Meal	32 (56.1)	43 (86.0)	0.0008
Rotavirus Vaccination (%)			0.3244
Yes	51 (89.5)	50 (100)	
No	1 (1.7)	0 (0)	
Missing *	5 (8.8)	-	

AGE, acute gastroenteritis; SD, standard deviation; %, percentage; *p*-values represent χ2 test of proportions (categorical variables) and Wilcoxon rank sum test (numerical variables); -, no count data; *, missing data were excluded from statistical analysis.

## Data Availability

Raw sequence files and metadata are available from Qiita (Study ID: 14757). Codes used to analyse the data are available at: https://github.com/ekquaye/Diarrhoea-and-Faecal-Microbiome (accessed on 3 August 2022).

## Supplementary Materials

The following are available online at https://www.mdpi.com/article/10.3390/ijms24043607/s1: Figure S1: Variable and balance selection using Selbal; Table S1: Simple linear model summary of alpha diversity estimates; Table S2: ADONIS PERMANOVA test summary for beta diversity metrics; Table S3: Core genera (Venn diagram); Table S4: Correlation network modules and membership across all groups, AGE cases, and healthy controls.
