# Supplementary material for: Altered Faecal Microbiota Composition and Structure of Ghanaian Children with Acute Gastroenteritis
Source: Int J Mol Sci. 2023 Feb 10;24(4):3607. doi: 10.3390/ijms24043607 (PMC9962333; doi:10.3390/ijms24043607)
Supplement: Supplementary file 1 [file ijms-24-03607-s001.zip › ijms-2133950-supplementary.pdf]

Supplementary Materials

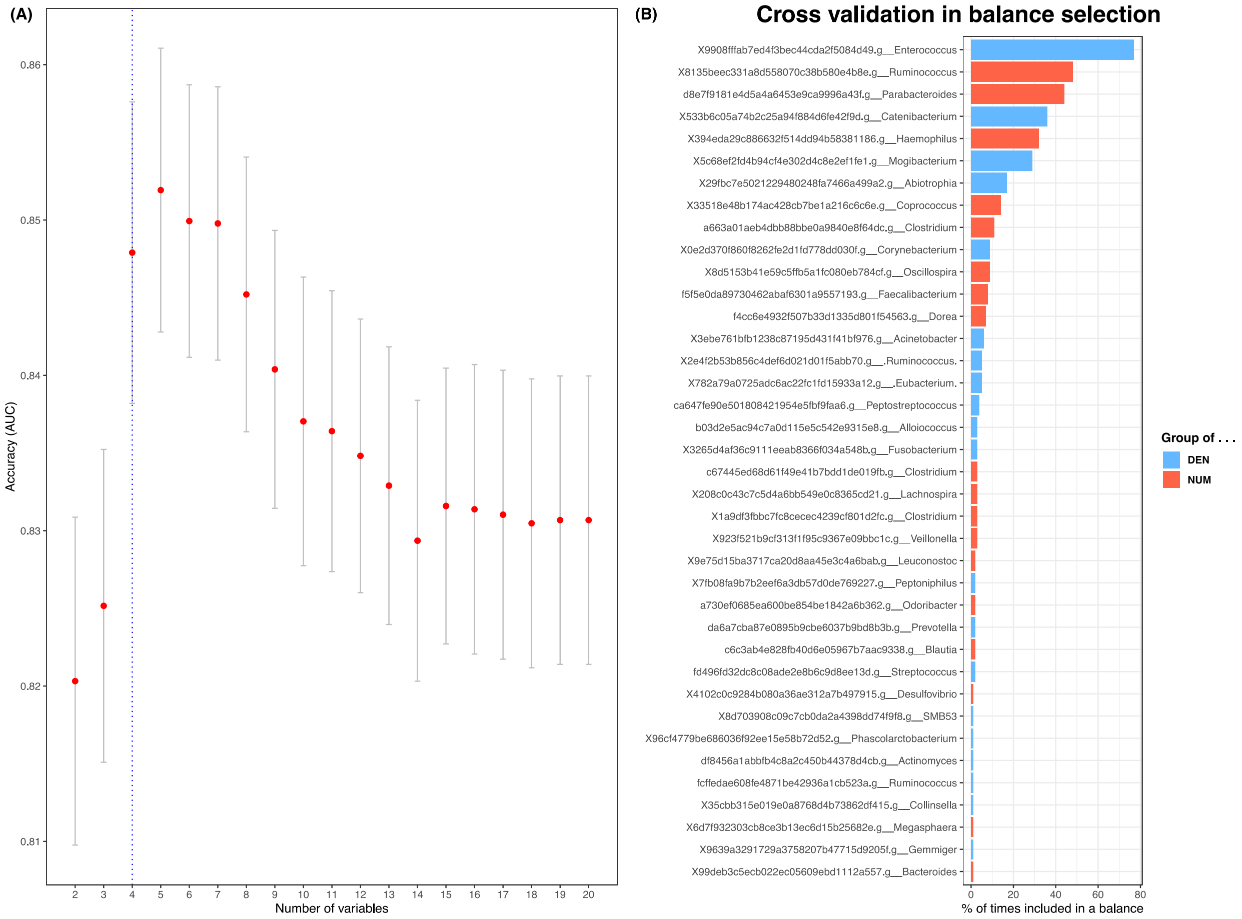

Figure S1. Variable and balance selection using Selbal. (A) Mean AUROC score based on the number of components in the cross-validation (CV) process. Four optimal number of components were included according to the "1se rule", as highlighted with the vertical dashed line. (B) Bar plot of the frequency of the variables selected in the CV process. The colour represents if the variable was selected as the numerator (red) or denominator (blue) of the balance. *Alloiococcus* = *Dolosigranulum*.

Table S1. Simple linear model summary of alpha diversity estimates.

	**Model A**		**Model B**		**Model C**	
	β Estimate	*p*-value	β Estimate	*p*-value	β Estimate	*p*-value
**Observed**						
Age	1.25	5.74e-04	0.67	0.13	0.72	0.13
Breastfeeding	-	-	-30.39	0.03	-39.18	0.03
Artificial milk	-	-	-	-	-6.35	0.77
Family meal	-	-	-	-	-19.59	0.30
Formula	-	-	-	-	-37.71	0.36
F-statistic	36.79	8.79e-13	27.02	6.07e-13	13.93	1.98e-11
Adjusted R-squared	0.41	-	0.43	-	0.43	-
**Shannon**						
Age	0.01	0.02	7.25e-3	0.30	5.80e-3	0.45
Breastfeeding	-	-	-0.33	0.15	-0.46	0.11
Artificial milk	-	-	-	-	-0.23	0.52
Family meal	-	-	-	-	-0.18	0.55
Formula	-	-	-	-	-0.49	0.47
F-statistic	45.06	8.72e-15	31.1	2.33e-14	15.58	1.63e-12
Adjusted R-squared	0.46	-	0.46	-	0.45	-
**PD**						
Age	0.06	6.42e-04	0.04	0.10	0.04	0.10
Breastfeeding	-	-	-1.31	0.06	-1.72	0.05
Artificial milk	-	-	-	-	-0.36	0.74
Family meal	-	-	-	-	-0.90	0.33
Formula	-	-	-	-	-1.10	0.59
F-statistic	33.56	5.99e-12	24.14	6.95e-12	12.19	3.17e-10
Adjusted R-squared	0.38	-	0.40	-	0.39	-

-, not applicable; covariates included in the linear models were as follows: model A (age only), model B (age, breastfeeding), and model C (age, breastfeeding, artificial milk, family meal, and formula)

Table S2. ADONIS PERMANOVA test summary for beta diversity metrics.

	**F-Model**	R^2	***p*-value**
**Bray-Curtis**			
Status	10.81	0.09	0.001
Age	3.01	0.03	0.003
Gender	0.74	0.00	0.739
Breastfeeding	2.21	0.02	0.010
Family meal	1.52	0.01	0.087
Artificial milk	1.22	0.01	0.242
Formula	1.17	0.00	0.243
**Weighted UniFrac**			
Status	25.50	0.19	0.001
Age	5.23	0.04	0.002
Gender	0.82	0.00	0.486
Breastfeeding	4.33	0.03	0.004
Family meal	0.72	0.00	0.593
Artificial milk	0.64	0.00	0.642
Formula	0.81	0.00	0.546
**Unweighted UniFrac**			
Status	15.19	0.12	0.001
Age	3.88	0.03	0.001
Gender	0.62	0.00	0.912
Breastfeeding	1.84	0.02	0.053
Family meal	1.34	0.01	0.162
Artificial milk	1.07	0.00	0.313
Formula	0.77	0.00	0.709

Table S3. Core genera (Venn diagram).

Group	Genera
AGE cases	*Actinomyces*<br>*Atopobium*<br>*Bifidobacterium*<br>*Blautia*<br>*Enterococcus*<br>*Granulicatella*<br>*Ligilactobacillus*<br>*Rothia*<br>*Streptococcus*<br>*Veillonella*
Healthy Controls	*[Eubacterium]*<br>*[Ruminococcus]*<br>*Actinomyces*<br>*Anaerostipes*<br>*Bacteroides*<br>*Bifidobacterium*<br>*Blautia*<br>*Clostridium*<br>*Clostridium*<br>*Clostridium*<br>*Collinsella*<br>*Coprococcus*<br>*Dialister*<br>*Dorea*<br>*Enterococcus*<br>*Faecalibacterium*<br>*Gemmiger*<br>*Granulicatella*<br>*Haemophilus*<br>*Lachnospira*<br>*Ligilactobacillus*<br>*Odoribacter*<br>*Oscillospira*<br>*Parabacteroides*<br>*Prevotella*<br>*Roseburia*<br>*Ruminococcus*<br>*Streptococcus*<br>*Sutterella*<br>*Veillonella*

Table S4. Correlation network modules and membership across all groups, AGE cases, and healthy controls.

Group	Module	Co-occurring Genus-level features
All groups	Module_0	Bacteria;Bacillota;Clostridia;Clostridiales;Lachnospiraceae;[Ruminococcus];NA
		Bacteria;Bacillota;Clostridia;Clostridiales;Lachnospiraceae;Coprococcus;NA
		Bacteria;Bacillota;Erysipelotrichi;Erysipelotrichales;Erysipelotrichaceae;[Eubacterium];NA
		Bacteria;Bacillota;Clostridia;Clostridiales;Ruminococcaceae;Ruminococcus;NA
		Bacteria;Bacillota;Clostridia;Clostridiales;Ruminococcaceae;Oscillospira;NA
		Bacteria;Bacteroidota;Bacteroidia;Bacteroidales;Bacteroidaceae;Bacteroides;NA
		Bacteria;Bacillota;Clostridia;Clostridiales;Lachnospiraceae;Blautia;NA
		Bacteria;Bacillota;Clostridia;Clostridiales;Lachnospiraceae;Dorea;NA
		Bacteria;Bacillota;Clostridia;Clostridiales;Ruminococcaceae;Faecalibacterium;NA
	Module_1	Bacteria;Actinomycetota;Actinobacteria;Actinomycetales;Micrococcaceae;Rothia;NA
		Bacteria;Bacillota;Bacilli;Lactobacillales;Carnobacteriaceae;Granulicatella;NA
		Bacteria;Actinomycetota;Coriobacteriia;Coriobacteriales;Coriobacteriaceae;Atopobium;NA
		Bacteria;Actinomycetota;Actinobacteria;Actinomycetales;Actinomycetaceae;Actinomyces;NA
		Bacteria;Bacillota;Bacilli;Lactobacillales;Streptococcaceae;Streptococcus;NA
	Module_2	Bacteria;Bacillota;Clostridia;Clostridiales;Lachnospiraceae;Lachnospira;NA

		Bacteria;Bacteroidota;Bacteroidia;Bacteroidales;Porphyromonadaceae;Parabacteroides;NA
		Bacteria;Pseudomonadota;Betaproteobacteria;Burkholderiales;Alcaligenaceae;Sutterella;NA
		Bacteria;Bacillota;Clostridia;Clostridiales;Lachnospiraceae;Roseburia;NA
	Module_3	Bacteria;Bacillota;Clostridia;Clostridiales;[Tissierellaceae];Finegoldia;NA
		Bacteria;Bacillota;Clostridia;Clostridiales;[Tissierellaceae];Peptoniphilus;NA
		Bacteria;Bacillota;Clostridia;Clostridiales;[Tissierellaceae];Anaerococcus;NA
	Module_4	Bacteria;Bacillota;Bacilli;Bacillales;Planococcaceae;Sporosarcina;NA
		Bacteria;Bacillota;Bacilli;Lactobacillales;Enterococcaceae;Vagococcus;NA
		Bacteria;Bacillota;Bacilli;Bacillales;Planococcaceae;Solibacillus;NA
	Module_5	Bacteria;Bacillota;Clostridia;Clostridiales;Lachnospiraceae;Oribacterium;NA
		Bacteria;Bacillota;Clostridia;Clostridiales;Lachnospiraceae;Moryella;NA
		Bacteria;Bacillota;Clostridia;Clostridiales;Lachnospiraceae;Lachnoanaerobaculum;NA
	Module_6	Bacteria;Bacillota;Erysipelotrichi;Erysipelotrichales;Erysipelotrichaceae;Clostridium;NA
		Bacteria;Bacillota;Clostridia;Clostridiales;Lachnospiraceae;Clostridium;NA
	Module_7	Bacteria;Bacillota;Bacilli;Turicibacterales;Turicibacteraceae;Turicibacter;NA
		Bacteria;Bacillota;Clostridia;Clostridiales;Clostridiaceae;SMB53;NA
	Module_8	Bacteria;Spirochaetota;[Brachyspirae];[Brachyspirales];Brachyspiraceae;Brachyspira;NA

		Bacteria;Pseudomonadota;Epsilonproteobacteria;Campylobacterales;Campylobacteraceae;Campylobacter;<br>NA
	Module_9	Bacteria;Actinomycetota;Actinobacteria;Actinomycetales;Corynebacteriaceae;Corynebacterium;NA
		Bacteria;Bacillota;Bacilli;Bacillales;Staphylococcaceae;Staphylococcus;NA
	Module_10	Bacteria;Bacillota;Clostridia;Clostridiales;Ruminococcaceae;Gemmiger;NA
		Bacteria;Bacteroidota;Bacteroidia;Bacteroidales;[Odoribacteraceae];Odoribacter;NA
	Module_11	Bacteria;Actinomycetota;Actinobacteria;Bifidobacteriales;Bifidobacteriaceae;Bifidobacterium;NA
		Bacteria;Bacillota;Bacilli;Lactobacillales;Lactobacillaceae;Lactobacillus;NA
	Module_12	Bacteria;Pseudomonadota;Gammaproteobacteria;Pasteurellales;Pasteurellaceae;Haemophilus;NA
		Bacteria;Bacillota;Clostridia;Clostridiales;Veillonellaceae;Veillonella;NA
	Module_13	Bacteria;Verrucomicrobiota;Verrucomicrobiae;Verrucomicrobiales;Verrucomicrobiaceae;Akkermansia;NA
		Bacteria;Bacillota;Clostridia;Clostridiales;Lachnospiraceae;Ruminococcus;NA
	Module_14	Archaea;Euryarchaeota;Methanobacteria;Methanobacteriales;Methanobacteriaceae;Methanobrevibacter;NA
		Archaea;Euryarchaeota;Methanobacteria;Methanobacteriales;Methanobacteriaceae;Methanosphaera;NA
	Module_15	Bacteria;Bacillota;Clostridia;Clostridiales;Veillonellaceae;Dialister;NA
		Bacteria;Bacteroidota;Bacteroidia;Bacteroidales;Prevotellaceae;Prevotella;NA
	Module_16	Bacteria;Bacillota;Clostridia;Clostridiales;[Tissierellaceae];Tissierella_Soehngenia;NA

		Bacteria;Bacillota;Clostridia;Clostridiales;Peptostreptococcaceae;Clostridium;NA
	Module_17	Bacteria;Pseudomonadota;Deltaproteobacteria;Desulfovibrionales;Desulfovibrionaceae;Bilophila;NA
		Bacteria;Bacillota;Clostridia;Clostridiales;Veillonellaceae;Phascolarctobacterium;NA
	Module_18	Bacteria;Bacteroidota;Bacteroidia;Bacteroidales;[Paraprevotellaceae];[Prevotella];NA
		Bacteria;Pseudomonadota;Gammaproteobacteria;Aeromonadales;Succinivibrionaceae;Succinivibrio;NA

Table S4. Cont.

Group	Module	Co-occurring Genus-level features
AGE cases	Module_0	Bacteria;Bacillota;Clostridia;Clostridiales;Lachnospiraceae;[Ruminococcus];NA
		Bacteria;Bacillota;Clostridia;Clostridiales;Lachnospiraceae;Coprococcus;NA
		Bacteria;Bacillota;Erysipelotrichi;Erysipelotrichales;Erysipelotrichaceae;Catenibacterium;NA
		Bacteria;Bacillota;Erysipelotrichi;Erysipelotrichales;Erysipelotrichaceae;[Eubacterium];NA
		Bacteria;Bacillota;Clostridia;Clostridiales;Ruminococcaceae;Gemmiger;NA
		Bacteria;Bacillota;Clostridia;Clostridiales;Lachnospiraceae;Blautia;NA
		Bacteria;Bacillota;Clostridia;Clostridiales;Lachnospiraceae;Dorea;NA
	Module_1	Bacteria;Bacillota;Clostridia;Clostridiales;[Tissierellaceae];Finegoldia;NA
		Bacteria;Bacillota;Clostridia;Clostridiales;[Tissierellaceae];Peptoniphilus;NA
		Bacteria;Bacillota;Clostridia;Clostridiales;[Tissierellaceae];Anaerococcus;NA
		Bacteria;Bacillota;Clostridia;Clostridiales;Peptostreptococcaceae;Peptostreptococcus;NA
	Module_2	Bacteria;Bacillota;Bacilli;Lactobacillales;Carnobacteriaceae;Granulicatella;NA
		Bacteria;Actinomycetota;Coriobacteriia;Coriobacteriales;Coriobacteriaceae;Atopobium;NA
		Bacteria;Actinomycetota;Actinobacteria;Actinomycetales;Actinomycetaceae;Actinomyces;NA
	Module_3	Bacteria;Bacillota;Bacilli;Bacillales;Planococcaceae;Sporosarcina;NA
		Bacteria;Pseudomonadota;Alphaproteobacteria;Rhizobiales;Brucellaceae;Pseudochrobactrum;NA

		Bacteria;Bacillota;Bacilli;Bacillales;Planococcaceae;Solibacillus;NA
	Module_4	Bacteria;Bacillota;Clostridia;Clostridiales;Veillonellaceae;Dialister;NA
		Bacteria;Bacteroidota;Bacteroidia;Bacteroidales;Prevotellaceae;Prevotella;NA
		Bacteria;Pseudomonadota;Betaproteobacteria;Burkholderiales;Alcaligenaceae;Sutterella;NA
	Module_5	Bacteria;Bacillota;Clostridia;Clostridiales;[Tissierellaceae];Tissierella_Soehngenia;NA
		Archaea;Euryarchaeota;Methanobacteria;Methanobacteriales;Methanobacteriaceae;Methanobrevibacter;NA
		Bacteria;Pseudomonadota;Gammaproteobacteria;Pseudomonadales;Moraxellaceae;Psychrobacter;NA
	Module_6	Bacteria;Actinomycetota;Actinobacteria;Actinomycetales;Micrococcaceae;Rothia;NA
		Bacteria;Bacillota;Bacilli;Lactobacillales;Streptococcaceae;Streptococcus;NA
	Module_7	Bacteria;Actinomycetota;Actinobacteria;Bifidobacteriales;Bifidobacteriaceae;Gardnerella;NA
		Bacteria;Bacillota;Bacilli;Lactobacillales;Aerococcaceae;Aerococcus;NA
	Module_8	Bacteria;Bacillota;Clostridia;Clostridiales;Lachnospiraceae;Moryella;NA
		Bacteria;Bacillota;Clostridia;Clostridiales;Lachnospiraceae;Lachnoanaerobaculum;NA
	Module_9	Bacteria;Spirochaetota;[Brachyspirae];[Brachyspirales];Brachyspiraceae;Brachyspira;NA
		Bacteria;Pseudomonadota;Epsilonproteobacteria;Campylobacterales;Campylobacteraceae;Campylobacter;NA
	Module_10	Bacteria;Bacteroidota;Bacteroidia;Bacteroidales;Bacteroidaceae;Bacteroides;NA

		Bacteria;Bacillota;Clostridia;Clostridiales;Ruminococcaceae;Faecalibacterium;NA
	Module_11	Bacteria;Actinomycetota;Actinobacteria;Bifidobacteriales;Bifidobacteriaceae;Bifidobacterium;NA
		Bacteria;Bacillota;Bacilli;Lactobacillales;Lactobacillaceae;Lactobacillus;NA
	Module_12	Bacteria;Pseudomonadota;Gammaproteobacteria;Enterobacteriales;Enterobacteriaceae;Morganella;NA
		Bacteria;Bacteroidota;Bacteroidia;Bacteroidales;Porphyromonadaceae;Parabacteroides;NA
	Module_13	Bacteria;Actinomycetota;Actinobacteria;Actinomycetales;Corynebacteriaceae;Corynebacterium;NA
		Bacteria;Bacillota;Bacilli;Bacillales;Staphylococcaceae;Staphylococcus;NA
	Module_14	Bacteria;Pseudomonadota;Gammaproteobacteria;Pasteurellales;Pasteurellaceae;Haemophilus;NA
		Bacteria;Bacillota;Clostridia;Clostridiales;Veillonellaceae;Veillonella;NA
	Module_15	Bacteria;Bacillota;Clostridia;Clostridiales;Ruminococcaceae;Oscillospira;NA
		Bacteria;Bacillota;Clostridia;Clostridiales;Lachnospiraceae;Roseburia;NA
	Module_16	Bacteria;Bacillota;Clostridia;Clostridiales;Lachnospiraceae;Lachnospira;NA
		Bacteria;Bacillota;Clostridia;Clostridiales;Veillonellaceae;Phascolarctobacterium;NA
	Module_17	Bacteria;Actinomycetota;Coriobacteriia;Coriobacteriales;Coriobacteriaceae;Enterococcus;NA
		Bacteria;Bacteroidota;Bacteroidia;Bacteroidales;Porphyromonadaceae;Petrimonas;NA

Table S4. Cont.

Group	Module	Co-occurring Genus-level features
Healthy	Module_0	Bacteria;Actinomycetota;Actinobacteria;Actinomycetales;Micrococcaceae;Rothia;NA
		Bacteria;Bacillota;Bacilli;Bacillales;Staphylococcaceae;Staphylococcus;NA
		Bacteria;Actinomycetota;Coriobacteriia;Coriobacteriales;Coriobacteriaceae;Atopobium;NA
		Bacteria;Bacillota;Bacilli;Lactobacillales;Leuconostocaceae;Weissella;NA
		Bacteria;Bacillota;Bacilli;Lactobacillales;Lactobacillaceae;Pediococcus;NA
		Bacteria;Bacillota;Bacilli;Lactobacillales;Streptococcaceae;Streptococcus;NA
	Module_1	Bacteria;Bacillota;Clostridia;Clostridiales;Lachnospiraceae;[Ruminococcus];NA
		Bacteria;Bacillota;Clostridia;Clostridiales;Lachnospiraceae;Coprococcus;NA
		Bacteria;Bacillota;Clostridia;Clostridiales;Lachnospiraceae;Blautia;NA
		Bacteria;Bacillota;Clostridia;Clostridiales;Lachnospiraceae;Dorea;NA
		Bacteria;Bacillota;Clostridia;Clostridiales;Ruminococcaceae;Faecalibacterium;NA
	Module_2	Bacteria;Pseudomonadota;Gammaproteobacteria;Pseudomonadales;Moraxellaceae;Acinetobacter;NA
		Bacteria;Pseudomonadota;Alphaproteobacteria;Rhodospirillales;Acetobacteraceae;Acetobacter;NA
		Bacteria;Bacillota;Bacilli;Lactobacillales;Leuconostocaceae;Leuconostoc;NA
		Bacteria;Actinomycetota;Actinobacteria;Actinomycetales;Actinomycetaceae;Actinomyces;NA
		Bacteria;Bacillota;Clostridia;Clostridiales;Lachnospiraceae;Lachnospira;NA

	Module_3	
		Bacteria;Bacillota;Clostridia;Clostridiales;Ruminococcaceae;Ruminococcus;NA
		Bacteria;Bacillota;Clostridia;Clostridiales;Lachnospiraceae;Roseburia;NA
	Module_4	Bacteria;Actinomycetota;Coriobacteriia;Coriobacteriales;Coriobacteriaceae;Eggerthella;NA
		Bacteria;Bacillota;Erysipelotrichi;Erysipelotrichales;Erysipelotrichaceae;Clostridium;NA
		Bacteria;Bacillota;Clostridia;Clostridiales;Lachnospiraceae;Clostridium;NA
	Module_5	Bacteria;Pseudomonadota;Deltaproteobacteria;Desulfovibrionales;Desulfovibrionaceae;Bilophila;NA
		Bacteria;Bacillota;Clostridia;Clostridiales;Veillonellaceae;Phascolarctobacterium;NA
		Bacteria;Pseudomonadota;Betaproteobacteria;Burkholderiales;Alcaligenaceae;Sutterella;NA
	Module_6	Bacteria;Pseudomonadota;Betaproteobacteria;Neisseriales;Neisseriaceae;Neisseria;NA
		Bacteria;Pseudomonadota;Gammaproteobacteria;Pseudomonadales;Pseudomonadaceae;Pseudomonas;NA
		Bacteria;Bacillota;Clostridia;Clostridiales;Lachnospiraceae;Moryella;NA
	Module_7	Bacteria;Bacillota;Clostridia;Clostridiales;Ruminococcaceae;Oscillospira;NA
		Bacteria;Bacteroidota;Bacteroidia;Bacteroidales;Bacteroidaceae;Bacteroides;NA
		Bacteria;Bacteroidota;Bacteroidia;Bacteroidales;Porphyromonadaceae;Parabacteroides;NA
	Module_8	Bacteria;Pseudomonadota;Gammaproteobacteria;Pasteurellales;Pasteurellaceae;Haemophilus;NA
		Bacteria;Bacillota;Clostridia;Clostridiales;Veillonellaceae;Veillonella;NA
		Bacteria;Bacteroidota;Bacteroidia;Bacteroidales;[Odoribacteraceae];Odoribacter;NA

	Module_9	
		Bacteria;Bacteroidota;Bacteroidia;Bacteroidales;Prevotellaceae;Prevotella;NA
	Module_10	Bacteria;Bacillota;Clostridia;Clostridiales;Lachnospiraceae;Anaerostipes;NA
		Bacteria;Bacillota;Clostridia;Clostridiales;Ruminococcaceae;Gemmiger;NA
	Module_11	Bacteria;Actinomycetota;Actinobacteria;Bifidobacteriales;Bifidobacteriaceae;Bifidobacterium;NA
		Bacteria;Bacillota;Bacilli;Lactobacillales;Lactobacillaceae;Lactobacillus;NA
	Module_12	Archaea;Euryarchaeota;Methanobacteria;Methanobacteriales;Methanobacteriaceae;Methanobrevibacter;NA
		Archaea;Euryarchaeota;Methanobacteria;Methanobacteriales;Methanobacteriaceae;Methanosphaera;NA
	Module_13	Bacteria;Pseudomonadota;Gammaproteobacteria;Aeromonadales;Succinivibrionaceae;Succinivibrio;NA
		Bacteria;Bacillota;Clostridia;Clostridiales;Lachnospiraceae;Butyrivibrio;NA
	Module_14	Bacteria;Bacillota;Bacilli;Turicibacterales;Turicibacteraceae;Turicibacter;NA
		Bacteria;Bacillota;Clostridia;Clostridiales;Clostridiaceae;SMB53;NA
	Module_15	Bacteria;Verrucomicrobiota;Verrucomicrobiae;Verrucomicrobiales;Verrucomicrobiaceae;Akkermansia;NA
		Bacteria;Bacillota;Clostridia;Clostridiales;Lachnospiraceae;Ruminococcus;NA
	Module_16	Bacteria;Actinomycetota;Actinobacteria;Actinomycetales;Corynebacteriaceae;Corynebacterium;NA
		Bacteria;Bacillota;Bacilli;Lactobacillales;Carnobacteriaceae;Granulicatella;NA
	Module_17	Bacteria;Pseudomonadota;Deltaproteobacteria;Desulfovibrionales;Desulfovibrionaceae;Desulfovibrio;NA

		Bacteria;Pseudomonadota;Betaproteobacteria;Burkholderiales;Oxalobacteraceae;Oxalobacter;NA
	Module_18	Bacteria;Bacillota;Erysipelotrichi;Erysipelotrichales;Erysipelotrichaceae;Catenibacterium;NA
		Bacteria;Bacteroidota;Bacteroidia;Bacteroidales;[Paraprevotellaceae];[Prevotella];NA
